# Supplementary material for: Sonodynamic Therapy for HER2+ Breast Cancer with Iodinated Heptamethine Cyanine–Trastuzumab Conjugate
Source: Int J Mol Sci. 2024 Sep 21;25(18):10137. doi: 10.3390/ijms251810137 (PMC11431973; doi:10.3390/ijms251810137)
Supplement: Supplementary file 1 [file ijms-25-10137-s001.zip › ijms-3195868-supplementary.pdf]

Supplementary Materials

Sonodynamic Therapy for HER2+ Breast Cancer with Iodinated Heptamethine Cyanine–Trastuzumab Conjugate

Dmytro Kobzev[1], Olga Semenova[1], Sarit Aviel-Ronen[2], Olesia Kulyk[1], Raanan Carmieli[3], Tajib Mirzabekov[4], Gary Gellerman[1], Leonid Patsenker[1,*]

[1] Department of Chemical Sciences, Faculty of Natural Sciences, Ariel University, Ariel, 40700, Israel; dmytrok@ariel.ac.il (DK); olgasem@ariel.ac.il (OS); olesiak@ariel.ac.il (OK); garyg@ariel.ac.il (GG); leonidpa@ariel.ac.il (LP);
[2] Adelson School of Medicine, Ariel University, Ariel 40700, Israel; Sheba Medical Center, Tel-Hashomer, Ramat Gan, Israel; saritav@ariel.ac.il
[3] Department of Chemical Research Support, Weizmann Institute of Science, Rehovot, 7610001, Israel; raanan.carmieli@weizmann.ac.il
[4] Biomirex, Inc., 27 Strathmore Road, Natick, MA 01760, USA; tmirzabekov@biomirex.com
[*] Correspondence: leonidpa@ariel.ac.il

*** Corresponding Author**

Leonid Patsenker – *Department of Chemical Sciences, Faculty of Natural Sciences, Ariel University, Ariel, 40700, Israel; Phone: +972 4038564; Email: leonidpa@ariel.ac.il*

Table of contents

1. Stability of dyes and Ab-conjugates upon light and ultrasound (US) irradiation 3

2. EPR spectra 5

3. Mice body weight 6

4. Calculations of the singlet oxygen generation rates (*r*) upon US irradiation 6

5. Histological images 7

6. NMR and HRMS data 7

1. Stability of dyes and Ab-conjugates upon light and ultrasound (US) irradiation

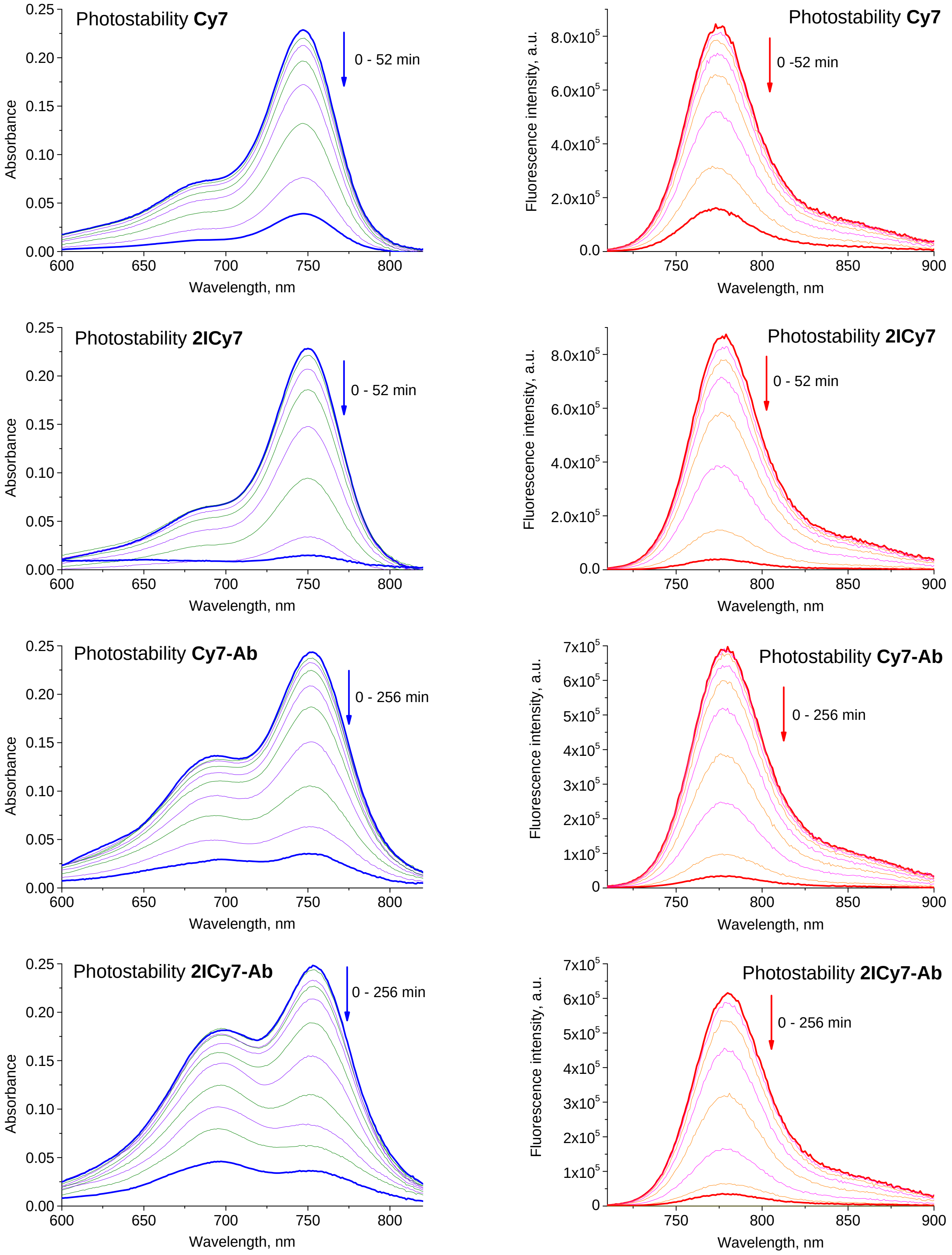

Fig. S1. Absorption and fluorescence spectra of **Cy7**, **2ICy7**, **Cy7-Ab**, and **2ICy7-Ab** (*c* ~ 1 μM) measured in 0.1 M PBS pH 7.4 over time upon light irradiation (730-nm LED, 8 mW/cm^2).

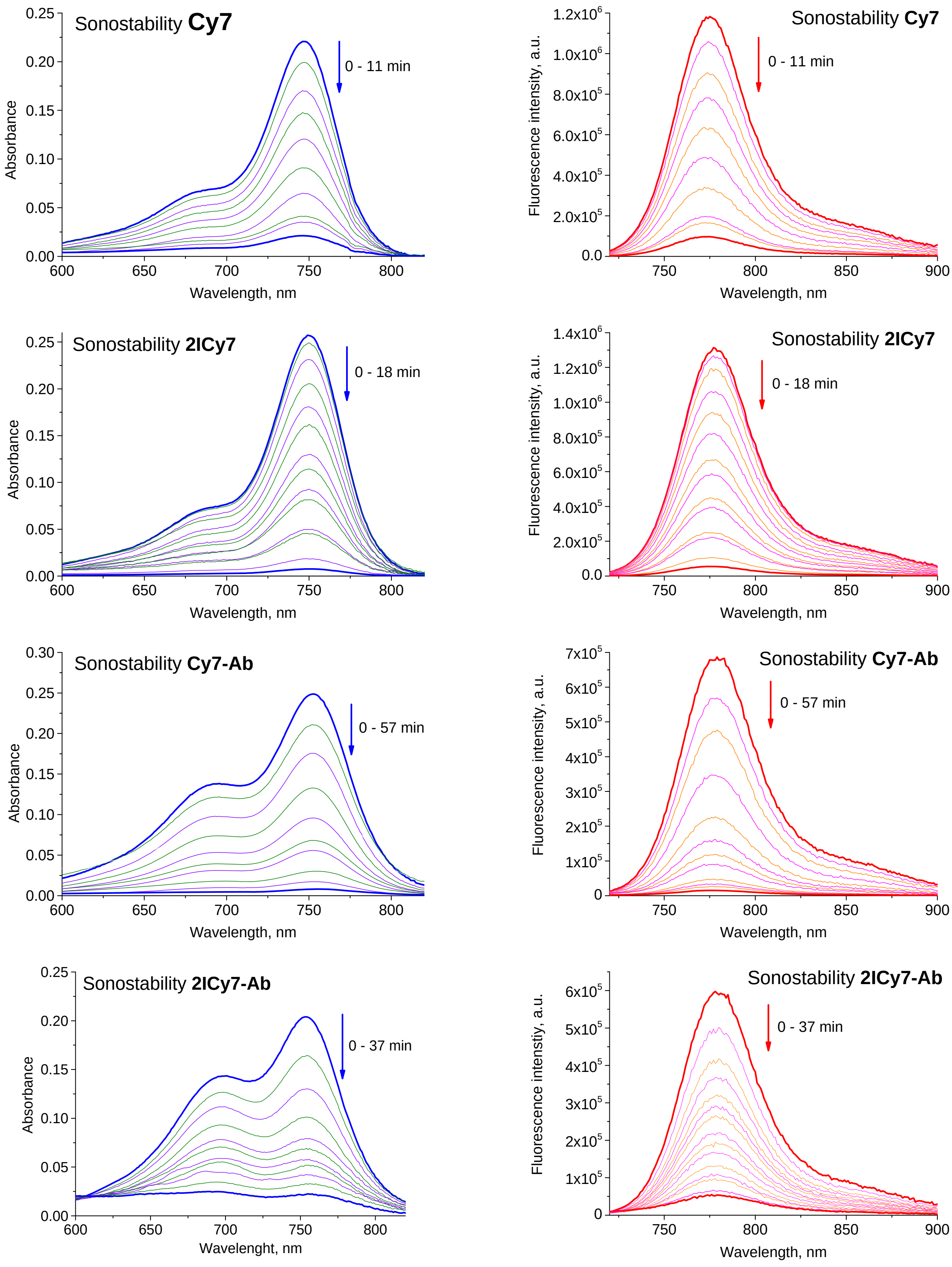

Fig. S2. Absorption and fluorescence spectra of **Cy7**, **2ICy7**, **Cy7-Ab**, and **2ICy7-Ab** (c ~ 1 μM) measured in 0.1 M PBS pH 7.4 over time upon US irradiation (f = 1 MHz, 700 mW/cm^2).

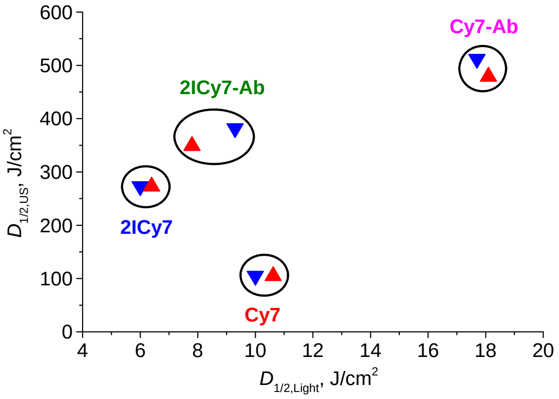

Fig. S3. Irradiation US dose ($D_{1/2,US}$) *vs.* light dose ($D_{1/2,Light}$) required for the 50% dye degradation measured by the decrease of the absorption (blue triangles) and emission (red triangles) bands. There is no clear correlation between $D_{1/2,US}$ and $D_{1/2,Light}$.

2. EPR spectra

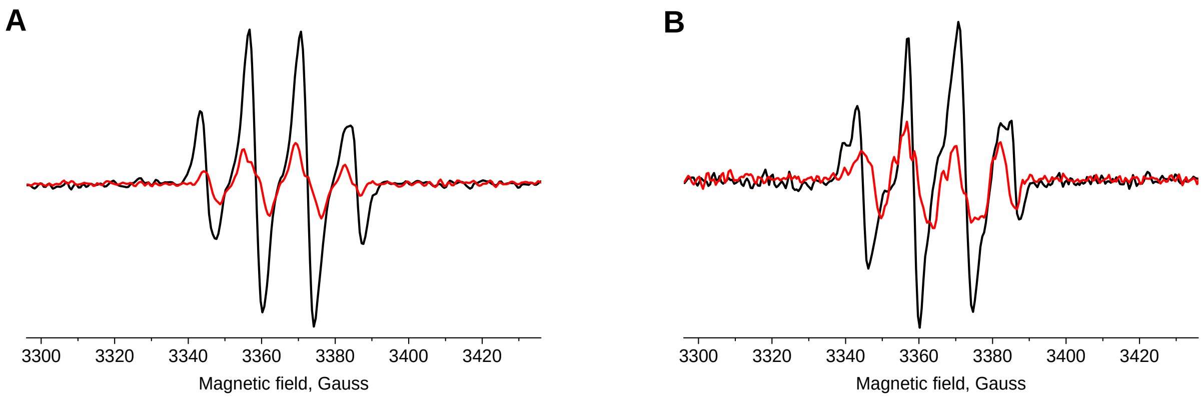

Fig. S4. EPR spectra of **2ICy7** (**A**) and **Cy7** (**B**) after 15 min of sonication without (black traces) and with DMSO (red traces).

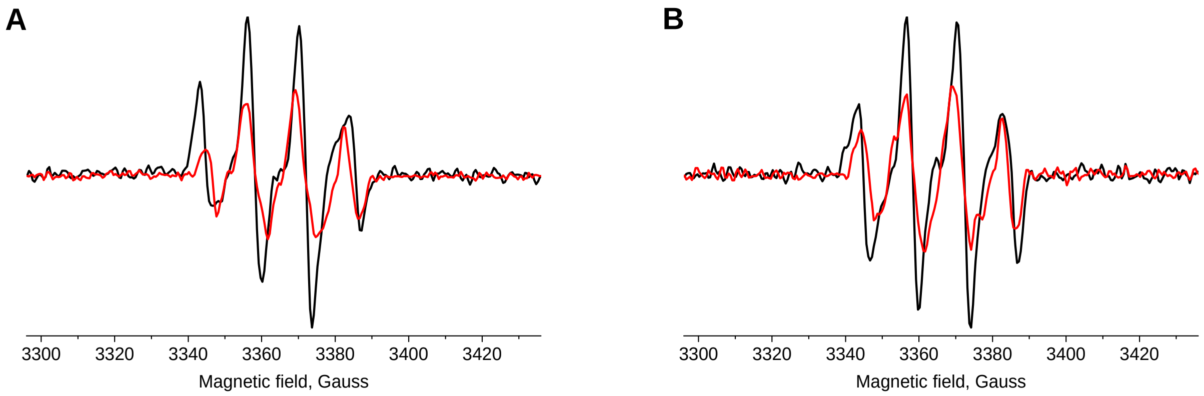

Fig. S5. EPR spectra of **2ICy7** (**A**) and **Cy7** (**B**) upon 30 min of light irradiation at 730 nm without (black traces) and with DMSO (red traces).

3. Mice body weight

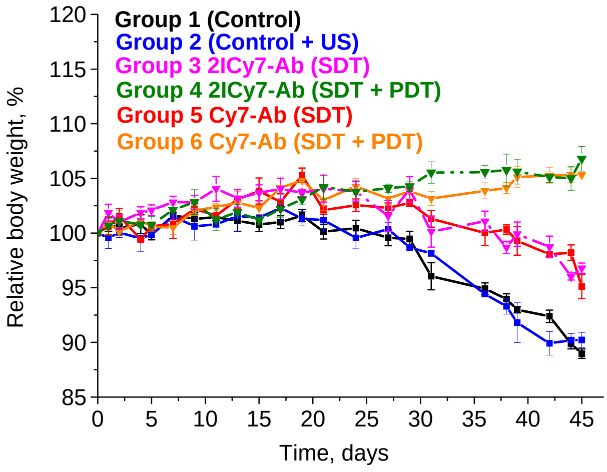

Fig. S6. Relative body weight curves. Body weight monitoring showed good tolerability of the treatment.

4. Calculations of the singlet oxygen generation rates (*r*) upon US irradiation

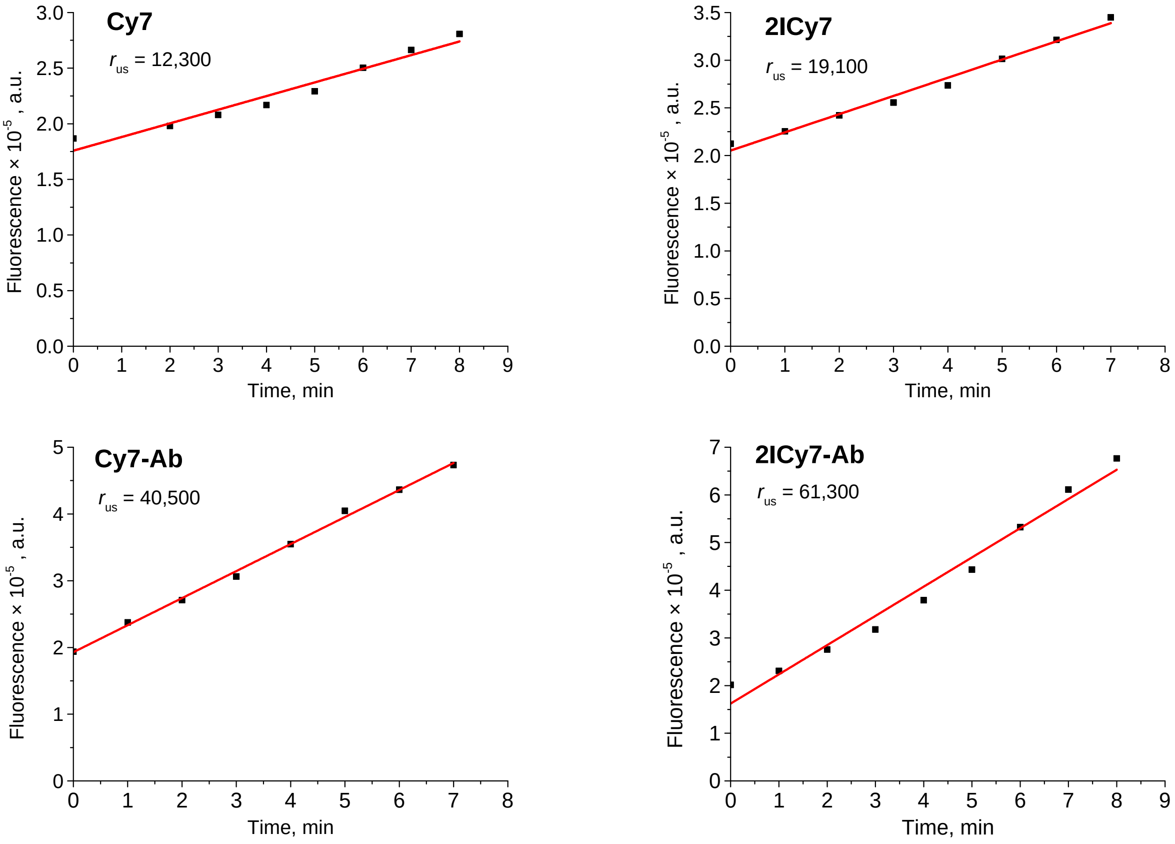

Fig. S7. The time-dependent emission intensities of **SOSG** measured at λ_{max} = 530 nm in 0.1 M PBS pH 7.4, which was used to calculate Φ_Δ for **Cy7** (**A**), **2ICy7** (**B**), **Cy7-Ab** (**C**), and **2ICy7-Ab** (**D**). Solutions of **SOSG** (*c* ~ 6 µM) containing corresponding sensitizers, **Cy7** (c_{Dye} ~ 2.3 µM), **2ICy7** (c_{Dye} ~ 1.7 µM), **Cy7-Ab** (c_{Dye} ~ 2.3 µM), or **2ICy7-Ab** (c_{Dye} ~ 1.3 µM), were sonicated (*f* = 1 MHz, 2 W/cm^2), the emission spectra were measured over time, the emission intensities at λ_{max} = 530 nm were fitted by a zero-order reaction rate function $y = a + r_{us} \cdot x$, and the reaction rates (r_{us}) were calculated.

5. Histological images

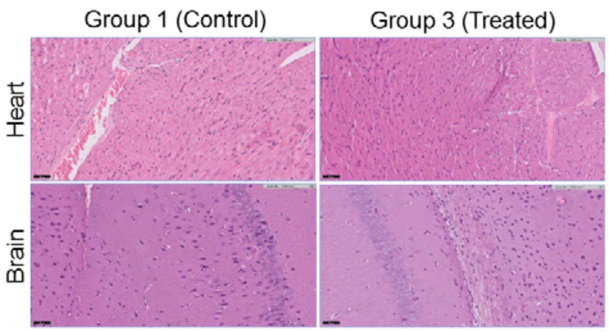

Fig. S8. Similarities in histopathological findings (H&E staining, magnification ×20) between control and SDT-treated (group 3) mice. The heart and brain were free of metastases in all the animals, both in the control and treated study groups.

6. NMR and HRMS data

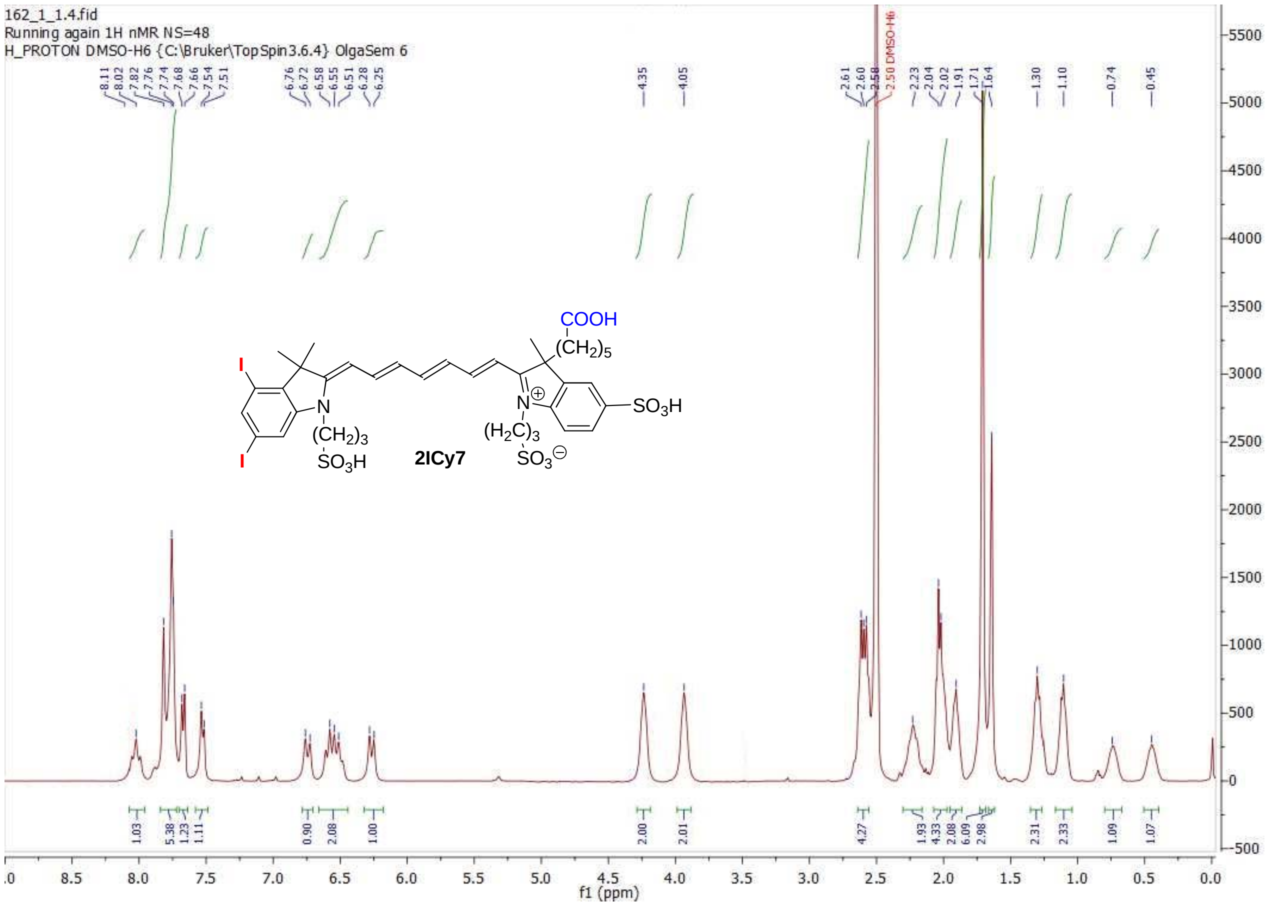

Fig. S9. ^{1}H NMR spectrum of **2ICy7** in DMSO-d_6.

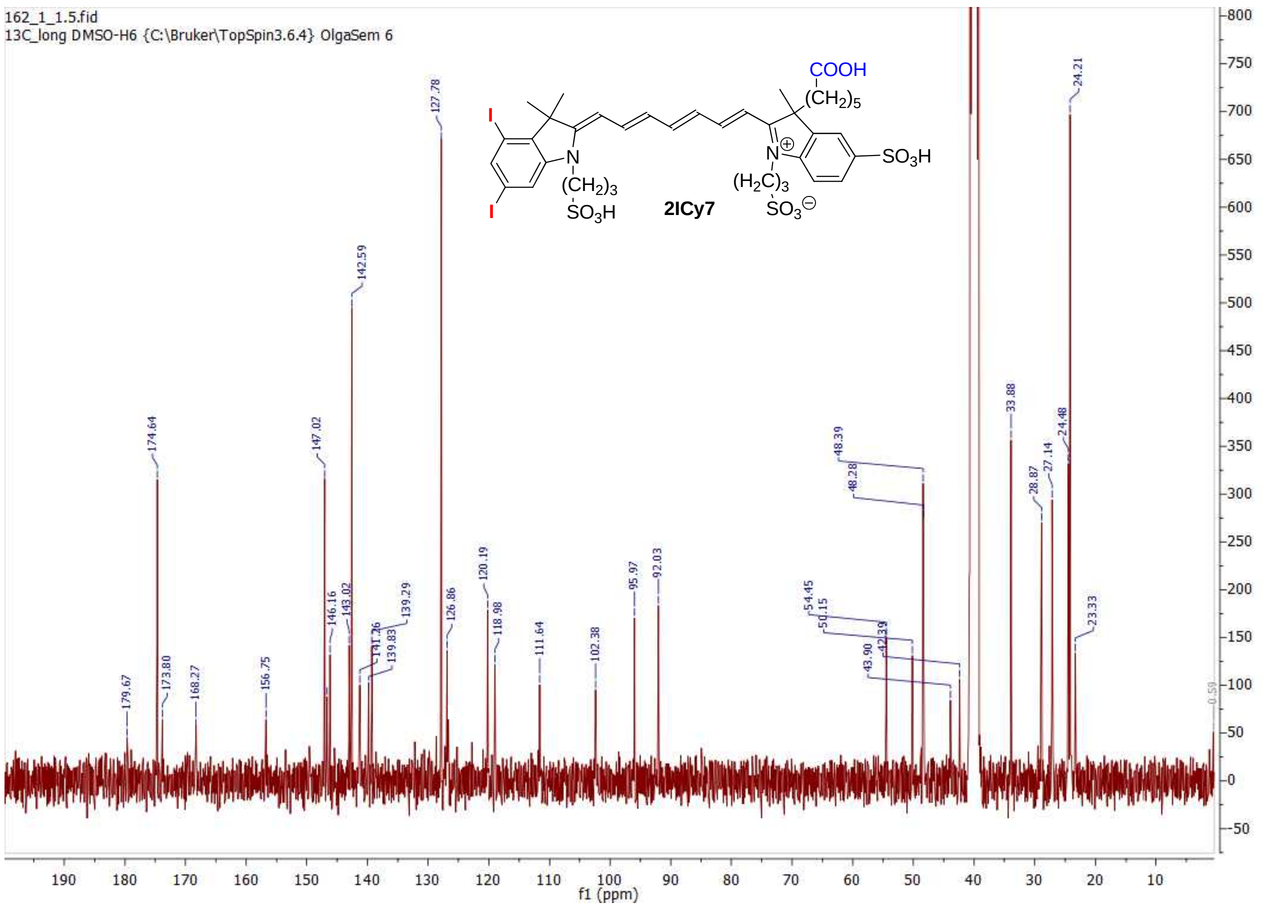

Fig. S10. ^{13}C NMR spectrum of **2ICy7** in DMSO-d_6.

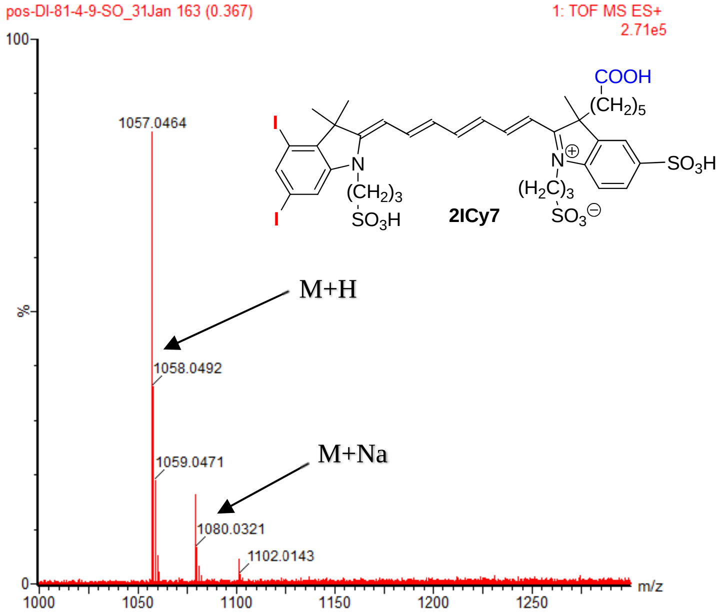

Fig. S11. HRMS spectrum of **2ICy7**.

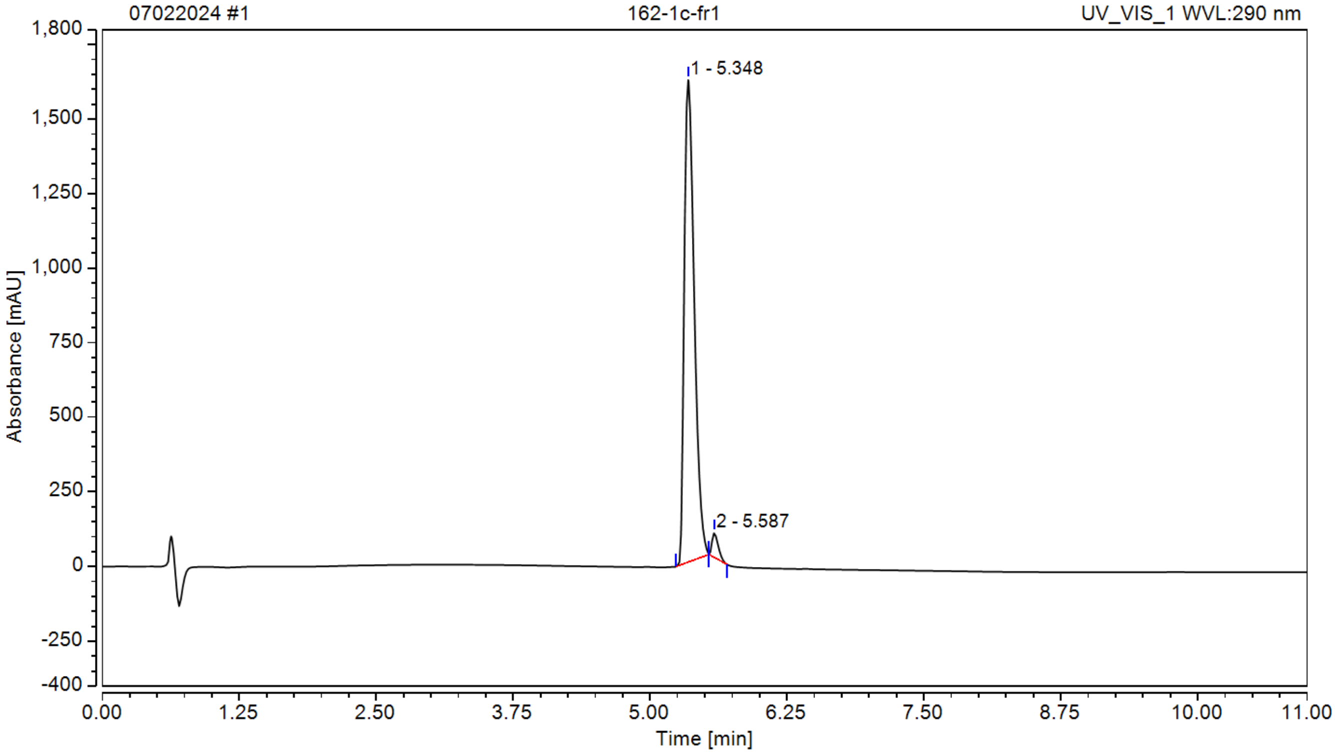

Fig. S12. LC-MS spectrum of **2ICy7**.

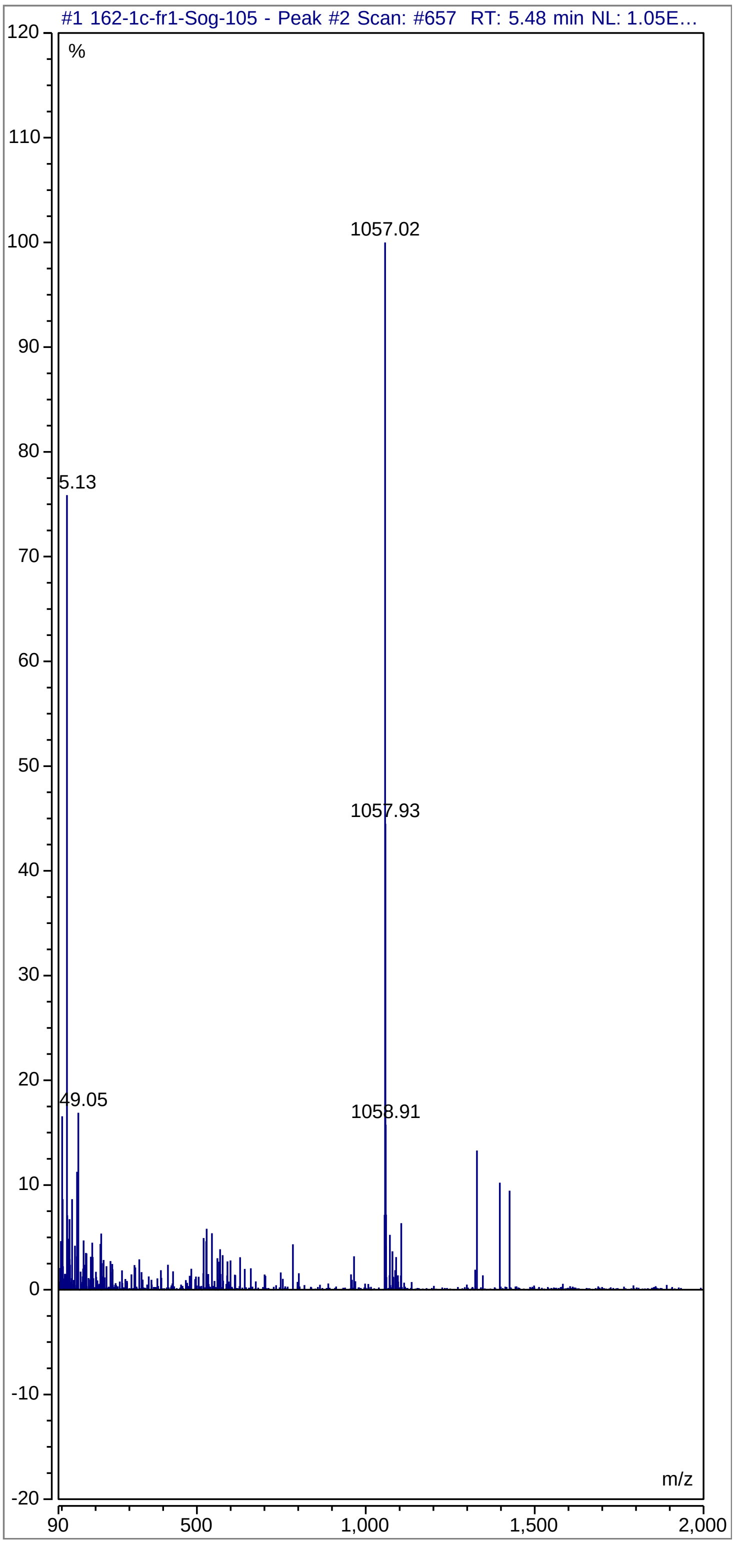

Fig. S13. Mass spectrum of **2ICy7** (for LC-MS 290 nm).
